# Genome-Wide Identification and Characterization of the AREB/ABF/ABI5 Subfamily Members from *Solanum tuberosum*

**DOI:** 10.3390/ijms20020311

**Published:** 2019-01-14

**Authors:** Tengfei Liu, Tingting Zhou, Meiting Lian, Tiantian Liu, Juan Hou, Raina Ijaz, Botao Song

**Affiliations:** 1 Key Laboratory of Horticultural Plant Biology, Ministry of Education, Huazhong Agricultural University, Wuhan 430070, China; hzauzsmj@gmail.com (T.L.); ttzh1989@126.com (T.Z.); Lmting2706@163.com (M.L.); 13296517556@163.com (T.L.); houjuanok@163.com (J.H.); rainaijaz@yahoo.com (R.I.); 2 Key Laboratory of Potato Biology and Biotechnology, Ministry of Agriculture and Rural Affairs, Huazhong Agricultural University, Wuhan 430070, China; 3 College of Horticulture, Henan Agricultural University, Zhengzhou 450002, China

**Keywords:** AREB/ABF, transcription factor, abscisic acid, osmotic stress, potato, gene regulation

## Abstract

Abscisic acid (ABA) plays crucial roles in plant development and adaption to environmental stresses. The ABA-responsive element binding protein/ABRE-binding factor and ABA INSENSITIVE 5 (AREB/ABF/ABI5) gene subfamily members, which belong to the basic domain/leucine zipper (bZIP) transcription factors family, participate in the ABA-mediated signaling pathway by regulating the expression of their target genes. However, information about potato (*Solanum tuberosum*) AREB/ABF/ABI5 subfamily members remains scarce. Here, seven putative *AREB/ABF/ABI5* members were identified in the potato genome. Sequences alignment revealed that these members shared high protein sequence similarity, especially in the bZIP region, indicating that they might possess overlapping roles in regulating gene expression. Subcellular localization analysis illustrated that all seven AREB/ABF/ABI5 members were localized in the nucleus. Transactivation activity assays in yeast demonstrated that these AREB/ABF/ABI5 members possessed distinct transcriptional activity. Electrophoretic mobility shift assays (EMSA) confirmed that all of these AREB/ABF/ABI5 members could have an affinity to ABRE in vitro. The expression patterns of these *AREB/ABF/ABI5* genes showed that they were in response to ABA or osmotic stresses in varying degrees. Moreover, most *AREB/ABF/ABI5* genes were induced during stolon swelling. Overall, these results provide the first comprehensive identification of the potato AREB/ABF/ABI5 subfamily and would facilitate further functional characterization of these subfamily members in future work.

## 1. Introduction

As sessile organisms, higher plants have evolved adaptive robustness to environmental variation. Abiotic stresses, such as drought and high-salinity, in which water availability is severely deficient, seriously threaten plant growth, survival, distribution, and productivity. Under stress conditions, numerous genes that serve the function of the stress tolerance response are induced in diverse plant species [1]. In-depth understanding of the mechanisms ensuring plant adaptation to stress can facilitate the development of tolerant varieties. Abscisic acid (ABA), one of five characteristic phytohormones, is involved in the regulation of numerous essential processes. Commonly, ABA is known as the “stress hormone” as it participates in adaptive responses to both abiotic and biotic stress, including drought, salt, cold, heat, water excess, UV-B radiation, heavy metal, and pathogens [2]. In addition to its function in stress responses, ABA regulates various developmental processes, such as embryo development, seed dormancy, germination, cotyledon greening, vegetative growth, and flowering [3,4].

ABA was discovered in the 1960s, however, the core ABA signaling pathway was only recently established. Core components of ABA signaling comprise ABA receptors PYRABACTIN RESISTANCE (PYR/PYL) [5], PROTEIN PHOSPHATASE 2C [6], SNF1-RELATED PROTEIN KINASE 2 (SnRK2) [7], ion channels [8,9,10], NADPH oxidases [11], and downstream transcription factors, such as the basic domain/leucine zipper (bZIP) class of ABA-responsive element binding protein/ABRE-binding factor (AREB/ABF) members (ABF1, AREB1/ABF2, AREB2/ABF4, and ABF3) [12]. Briefly, in the absence of ABA, PYR/PYLs are not bound to PP2Cs and PP2C activity is high, which prevents SnRK2 activation. In the presence of ABA, binding of ABA to PYR1/PYLs leads to inactivation of PP2C. The protein phosphatases seem to function as coreceptors and their inactivation launches SNF1-type kinase action which targets downstream transcription factors to regulate ABA-dependent gene expression and ion channels [4].

The ABA-responsive element (ABRE; PyACGTGG/TC), firstly identified by analyzing the promoters of ABA-inducible genes [13], is involved in mediating the induced expression of various genes in response to ABA. In addition, ABRE-like sequences, such as the G-box sequence and coupling element, CE3, hex3, and motif III have also been reported [14]. All divergent types of ABRE sequences are found to interact with AREB/ABF transcription factors at least in vitro [14]. The AREB/ABFs have been isolated by using ABRE as bait in yeast one-hybrid screening [15]. So far, nine *AREB/ABF* homologs-*AREB1/ABF2*, *AREB2/ABF4*, *AREB3/DPBF3*, *ABF1*, *ABF3/DPBF5*, *ABI5/DPBF1*, *EEL/DPBF4*, *DPBF2*, and *AT5G42910*-have been identified in the *Arabidopsis* genome [15,16,17,18,19]. Among them, AREB1/ABF2, AREB2/ABF4, ABF3, and ABF1 were reported to function predominantly in gene expression downstream of SnRK2 kinases in abscisic acid signaling in response to osmotic stress [12]. ABI5/DPBF1 has been shown to play a pivotal role in ABA-dependent postgerminative growth arrest [20,21]. However, the ABA signal pathways in other crop plants, such as potato, remain elusive.

Potato (*Solanum tuberosum* L.), the world’s third largest food crop, plays vital roles in food security, reducing poverty, and improving human nutrition. Nevertheless, the increasing abiotic stresses, caused by global climate change, are becoming a major threat to potato production. Increasing heat, drought, and salinity stresses will present enormous challenges to potato breeders to better understand the genes, traits, and management techniques that improve potato abiotic stresses adaptation [22].

Potato whole genome sequencing [23] allows researchers to identify gene families at a genome-wide level. Subsequently, in two separate studies, 438 NB-LRR (nucleotide binding-site leucine-rich repeat) genes and 435 NBS-encoding *R* genes were reported in the potato genome respectively [24,25]. Additionally, an increasing number of gene families were identified in the potato genome, such as 110 NAC gene family members [26], 41 aquaporin gene family members [27], 31 LEA gene family members [28], 35 Dof gene family members [29], 30 BBX gene family members [30], 155 ERF gene family members [31], and 124 bHLH gene family members [32]. However, the AREB/ABF/ABI5 subfamily has not been investigated in the potato with the exception of *StABF1*, which was reported to be induced by ABA, drought, salt stress, and cold [33], whereas the precise function of *StABF1* remains to be elucidated.

Considering the crucial roles played by the AREB/ABF/ABI5 subfamily in plants, genes encoding AREB/ABF/ABI5 members in potato were identified and characterized in the present study. Subcellular localization, transactivation activity, and DNA-binding activity analysis of the potato AREB/ABF/ABI5 members were performed. Stimulus-induced expression profiles were investigated under ABA and osmotic stresses treatment. The expression patterns of these *AREB/ABF/ABI5* in the stolon swelling process were also examined. Overall, this research would provide a solid basis for further precise functional characterization of each member in the subfamily and potato improvement by utilizing them.

## 2. Results

### 2.1. Identification and Gene Structure Analysis of the AREB/ABF/ABI5 Subfamily in Potato

**Figure 1:**
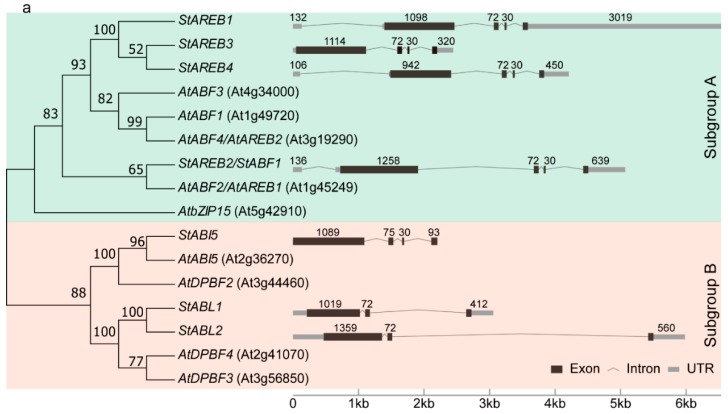

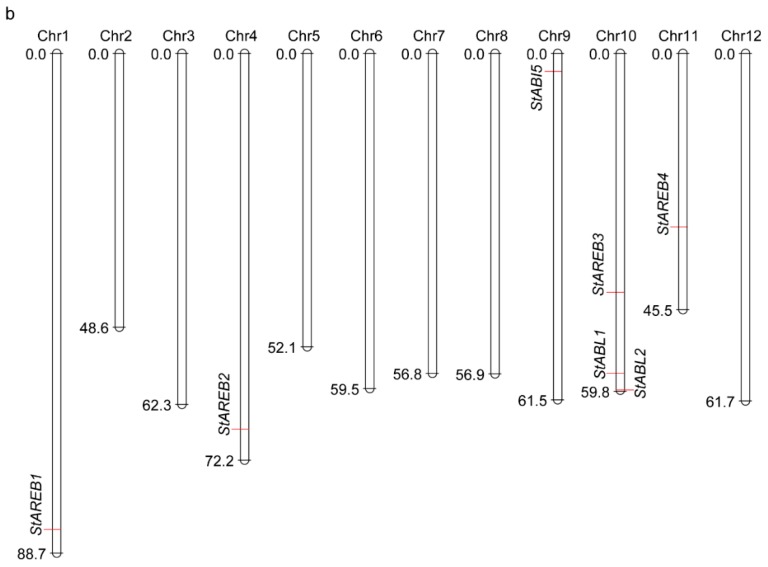
Sequence analysis and chromosomal localization of potato AREB/ABF/ABI5 members. (**a**) Neighbor-joining tree of potato and *Arabidopsis* AREB/ABF/ABI5 proteins and the gene structure of each potato *AREB/ABF/ABI5* member. The sizes (bp) of exons are as indicated. (**b**) The seven identified *AREB/ABF/ABI5* members were mapped on the four chromosomes.

To survey the *AREB/ABF/ABI5* subfamily in the potato genome, a genome-wide search against the PGSC database was performed via selecting the nine known *AREB/ABF/ABI5* homologs from *A. thaliana*. A total of seven distinct genes were identified as putative members of the potato *AREB/ABF/ABI5* subfamily. To verify and elucidate the relationships between potato and *A. thaliana* AREB/ABF/ABI5 members, a phylogenetic tree of AREB/ABF/ABI5 proteins was constructed. As illustrated in Figure 1a, these genes could be divided into two small subgroups. Four potato genes belonging to subgroup A were termed as StAREB, followed by the Arabic numbers 1–4, based on their relatively high similarities to *A. thaliana* AREBs and relatively linear order along chromosomes (Table 1, Figure 1). The gene PGSC0003DMG400002660, which shared the highest amino acid sequence homology with *Arabidopsis* AtABI5 was designated as StABI5. The other two members belonging to subgroup B, which had a close relationship with AtABI5 and StABI5, were named as StABL1 (ABI5-Like 1) and StABL2 (ABI5-Like 2), respectively (Table 1, Figure 1a). The chromosomal distributions of the seven genes were examined. These genes were found on 5 out of 12 chromosomes. *StAREB1*, *StAREB2*, *StABI5*, and *StAREB4* were localized on chromosome 1, 4, 9, and 11, respectively. Chromosome 10 contained three loci, including *StAREB3*, *StABL1*, and *StABL2* (Figure 1b).

To gain further insight into the potato *AREB/ABF/ABI5* subfamily, the intron-exon structure for the *AREB/ABF/ABI5* members was determined. It was found that *StAREB1*, *StAREB2*, and *StAREB4* possessed five exons, *StAREB3* and *StABI5* contained four exons, whereas *StABL1* and *StABL2* only comprised three exons (Figure 1b). Six genes contained an exon that was 72 bp long, while this conserved exon changed to 75 bp long in *StABI5* (Figure 1b). Five genes had a 30-bp exon adjacent to the conserved exon. The genomic sequence lengths of the *AREB/ABF/ABI5* genes ranged from 2204 to 6521 base pairs, indicating a high variability of sequence lengths among this subfamily (Figure 1b).

### 2.2. Sequence Analysis of Potato AREB/ABF/ABI5 Proteins

**Figure 2:**
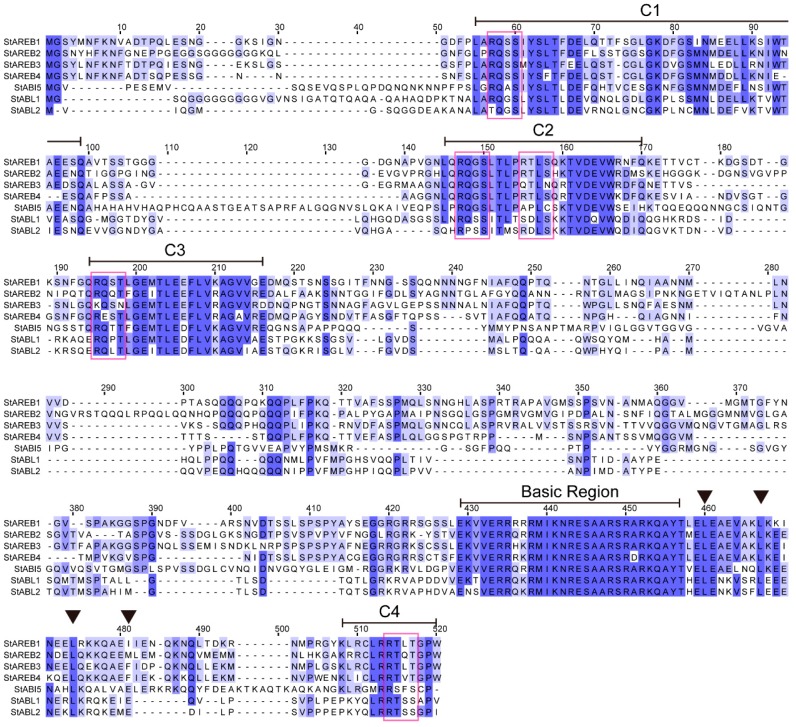
Multiple sequence alignments of potato AREB/ABF/ABI5 members. Identical and conserved residues are shaded in blue and light blue, respectively. The positions of the C1 to C4 conserved domains and of the basic region are indicated by lines above the protein sequences. Potential phosphoresidues (R-X-X-S/T) corresponding to the characterized phosphorylation sites in *Arabidopsis* AREB1 are indicated by red frames. The positions of the conserved Leu residues in the Leu zipper domain are indicated by a black triangle. Protein sequence alignments were performed by Jalview 2.10 (http://www.jalview.org/).

The full-length amino acid sequences of the encoded AREB/ABF/ABI5 members showed 30.40–70.15% sequence identity. The further analysis of these proteins by the Pfam database suggests that they all contain a conserved bZIP domain. Moreover, the amino acid sequence of the basic region is extremely conserved between the seven members of this subfamily (Figure 2). This region includes, in particular, the motifs M-I-K and Q-A-Y, which are reported to be specific for the *Arabidopsis* AREB/ABF/ABI5 members [18]. High conservation of this DNA binding region suggests that all members of this subfamily might recognize similar *cis* elements. As previously reported, three additional conserved domains are present in the N-terminus of the proteins, and a fourth is found at their C-terminal end [15,16,17]. The results of previous research suggest that activation of AREB/ABF/ABI5 requires ABA-dependent phosphorylation of multiple RXXS/T sites in the conserved region [34,35,36]. Amino acid sequence analysis of potato AREBs indicated that five RXXS/T sites distributed among C1 to C4 domains are conserved in most members (Figure 2), indicating that phosphorylation of the conserved Ser/Thr residues might account for regulation of these AREB/ABF/ABI5 members activations.

### 2.3. Subcellular Localization of Potato AREB/ABF/ABI5 Subfamily Members

**Figure 3:**
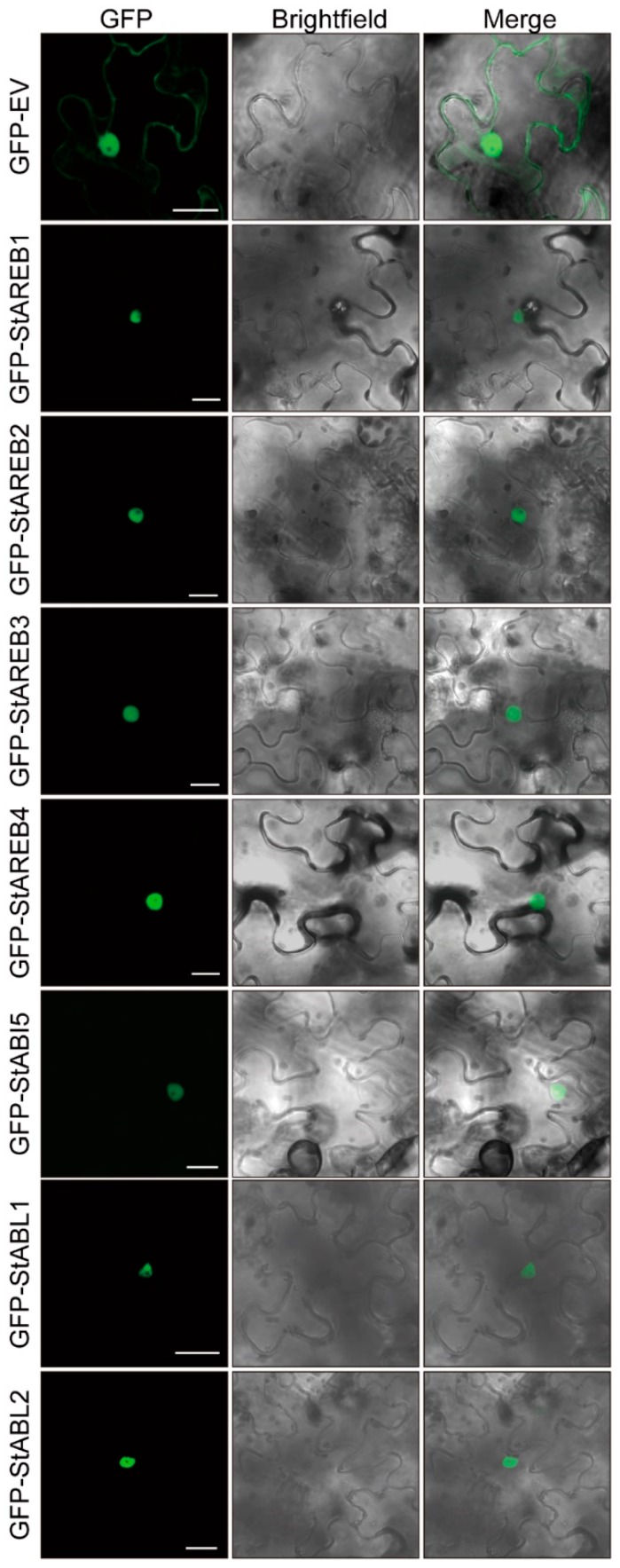
Subcellular localization analysis of potato AREB/ABF/ABI5 members. The GFP alone and GFP-fusion proteins were transiently expressed in *N. benthamiana* leaves via agroinfiltration and the images of epidermal cells were taken at 60 hpi by CLSM. Green color indicates that GFP alone is localized in both the cytoplasm and nucleus, AREB/ABF/ABI5 members are mainly localized in the nucleus. Bars = 20 μm.

The AREB/ABF/ABI5 members belong to the family of the bZIP transcript factors predicted to be localized in the nucleus (Table 1). To verify this, full-length CDS of these genes were fused to the C terminus of green fluorescent protein (GFP). The seven GFP-fusion proteins and GFP alone were transiently expressed in *N. benthamiana* by agroinfiltration. As illustrated in Figure 3, cells expressing GFP-fusion proteins showed that fluorescent signals were exclusively restricted to the nucleus, whereas the signal for cells expressing GFP alone was found in the nucleus and cytosol. These observations indicate that all seven AREB/ABF/ABI5 members are localized in the nucleus where they might regulate transcriptional events.

### 2.4. Transactivation Analysis of Potato AREB/ABF/ABI5 Members in Yeast

**Figure 4:**
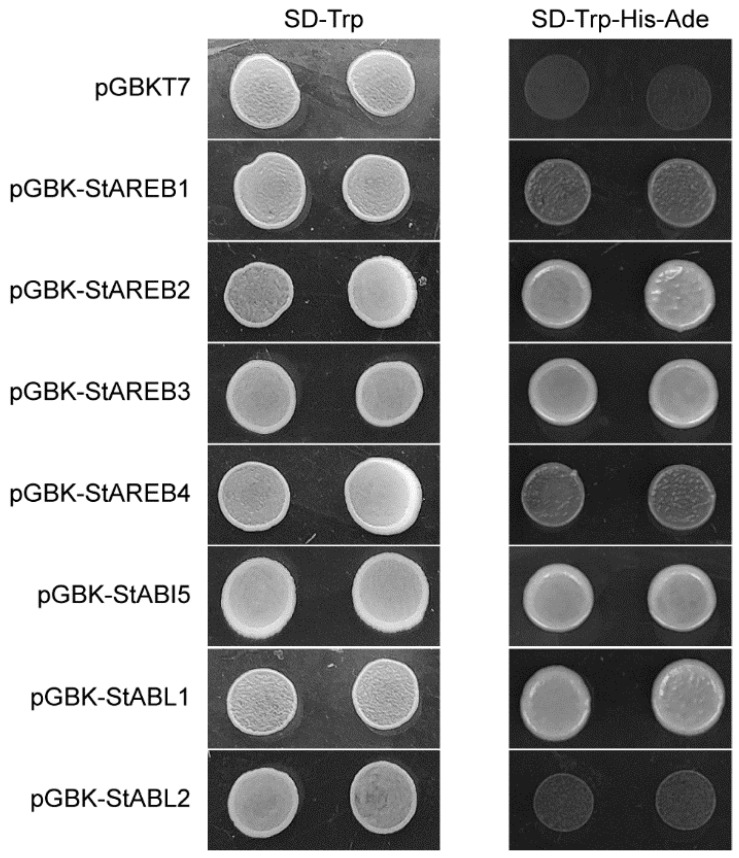
Transcriptional activation analysis of potato AREB/ABF/ABI5 members in yeast. The recombinant vectors were transferred into yeast strain AH109. The transcriptional activation ability of these proteins was analyzed by growing on SD/-Trp and SD/-Trp-His-Ade plates. The yeast strain AH109 harboring the pGBKT7 vector was used as a negative control.

To further characterize the function of the potato AREB/ABF/ABI5 members, a transcription activation assay was conducted in yeast. Full-length CDS of these *AREB* genes were fused in-frame with the GAL4 DNA-binding domain in the pGBKT7 vector, and the fusion constructs were then transformed into the yeast strain AH109. As shown in Figure 4, all the transformants grew normally on tryptophan-deficient medium (SD -Trp), while only transformants harboring pGBK-StAREB2, pGBK-StAREB3, pGBK-StABI5, and pGBK-StABL1 grew normally on medium lacking tryptophan, histidine, and adenine (SD -Trp-His-Ade). Transformants harboring pGBK-StAREB1 and pGBK-StAREB4 could also grow on SD -Trp-His-Ade but more weakly than the formers. Additionally, transformants harboring pGBK-StABL2 hardly grew on SD -Trp-His-Ade, as well as the negative control. Taken together, these results indicated that apart from StABL2, the other six StAREB/ABF/ABI5s possessed transactivation activity in yeast.

### 2.5. In Vitro DNA Binding Activity of Potato AREB/ABF/ABI5 Proteins

To obtain the recombinant AREB/ABF/ABI5 proteins, full-length CDS of these genes were fused in-frame with glutathione S- transferase (GST)-tag in the pET42b vector, and the fusion constructs were transformed into *Escherichia coli* strain *Rosetta*. However, except for StABL1 and StABL2, the other five proteins failed to purify in *E. coli*. To overcome the obstruction, the bZIP domains of these genes were truncated to fuse in-frame with GST-tag (Figure S1), resulting in a high yield of purified proteins (Figure S2). These purified full-length or truncated AREB/ABF/ABI5 proteins containing the bZIP domain were used for EMSA assays.

**Figure 5:**
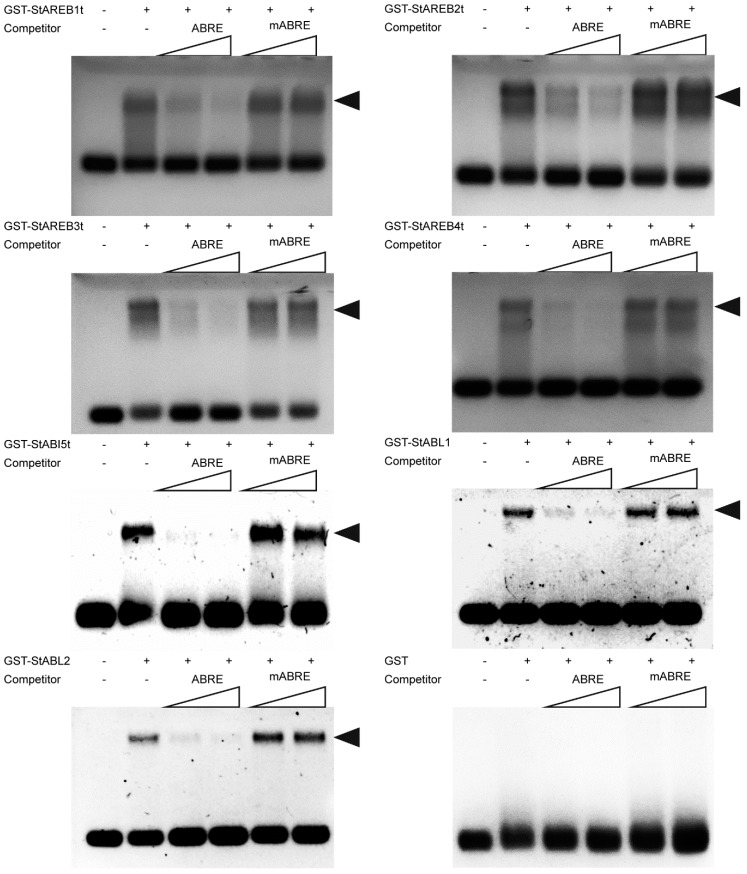
Electrophoretic mobility shift assay (EMSA). The oligonucleotide (ABRE) containing the Em1a element (GGACACGTGGCG) was employed as a FAM-labeled probe in a mobility shift assay. In each assay, 1 μg of purified recombinant full-length or truncated AREB/ABF/ABI5 was used. The ‘+’ and ‘−’ indicate the presence and absence of the indicated probe or protein. Lanes 3 and 4, and 5 and 6 represent a 50-fold and 100-fold molar excess of the specific unlabeled competitor (ABRE) or unlabeled mutated oligonucleotide (mABRE) competitor. Sequences of oligonucleotides are as follows: ABRE, aattccGGACACGTGGCGtaagct; mABRE, aattccGGACctacaGCCtaagct. Shifted bands are indicated by an arrowhead.

To test the in vitro DNA binding activity of AREB/ABF/ABI5 proteins, EMSA was performed by using recombinant proteins and a probe DNA containing ABRE. The EMSA assay results of these recombinant proteins are shown in Figure 5. No shifted band was detected with the GST negative control; however, a major shifted band was observed with all seven full-length or truncated AREB/ABF/ABI5 proteins. The addition of excess, increasing amounts of unlabeled probe DNA to the reaction mixtures gradually abolished the binding, whereas the same amount of a mutated oligonucleotide did not, revealing robust interactions between these recombinant proteins and ABRE. Thus, these results indicate that these full-length or truncated AREB/ABF/ABI5 proteins all exhibited sequence-specific binding activity to the ABRE in vitro.

### 2.6. Expression Patterns of the Potato AREB/ABF/ABI5 Members in Response to ABA and Osmotic Stresses

**Figure 6:**
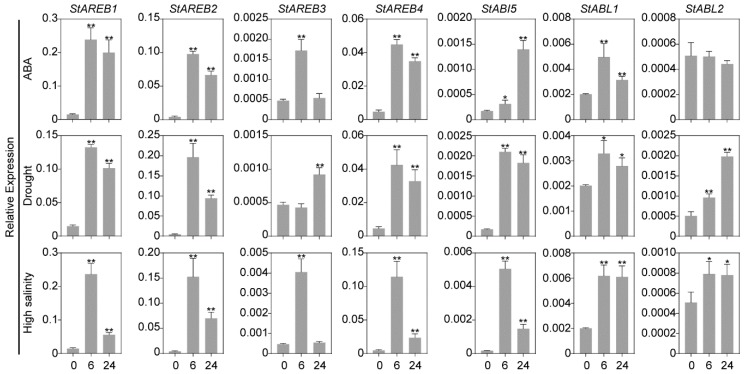
Expression profiles of potato *AREB/ABF/ABI5* members under treatments of ABA, dehydration, and high salinity. The relative expression levels are normalized to *ef1α*. ABA, dehydration, and high salinity represent the in vitro plants treated with 5 μM ABA, water deficit, and 125 mM NaCl. The numbers 0, 6, and 24 indicate the time (hour) after treatments. Error bars represent the standard deviation (SD) of three biological replicates. * and ** represents significant differences in *p* values < 0.5 and < 0.01, respectively.

*AREB/ABF/ABI5* members have been reported to be induced by ABA and osmotic stresses in several plant species [12,37,38,39,40]. To assess the ABA- and osmotic stresses-responsive expression of potato *AREB/ABF/ABI5* members, qRT-PCR analyses were performed. The expression profiles of these *genes* were measured after being challenged by ABA, drought, and high salinity. It was observed that except for *StABL2*, which showed to insensitive to ABA treatment, the other six *AREB/ABF/ABI5* members displayed varying responses upon ABA treatment. Among them, the expression of *StAREB1*, *StAREB2*, and *StAREB4* was dramatically induced at 6 h, with a slight decrease at 24 h (Figure 6). However, the responses of *StAREB3*, *StABI5*, and *StABL2* challenged with ABA were mild, as shown in Figure 6; the expression of *StABI5* was gradually increased, while the transcripts of *StAREB3* and *StABL2* were accumulated at 6h. Under dehydration and NaCl stress, the responses of *StAREB1, StAREB2*, and *StAREB4* were similar to that of ABA treatment, the other four *AREB/ABF/ABI5* members showed various up-regulated expression levels between 6 and 24 h of stress treatments (Figure 6). According to the qRT-PCR results, the potato *AREB/ABF/ABI5* members were notably affected and showed a partially different expression pattern in response to these abiotic stresses, indicating that they might play both overlapping and distinct roles in ABA signaling and osmotic stresses.

### 2.7. Expression of the Potato AREB/ABF/ABI5 Members in Distinct Stages of Stolon Swelling

**Figure 7:**
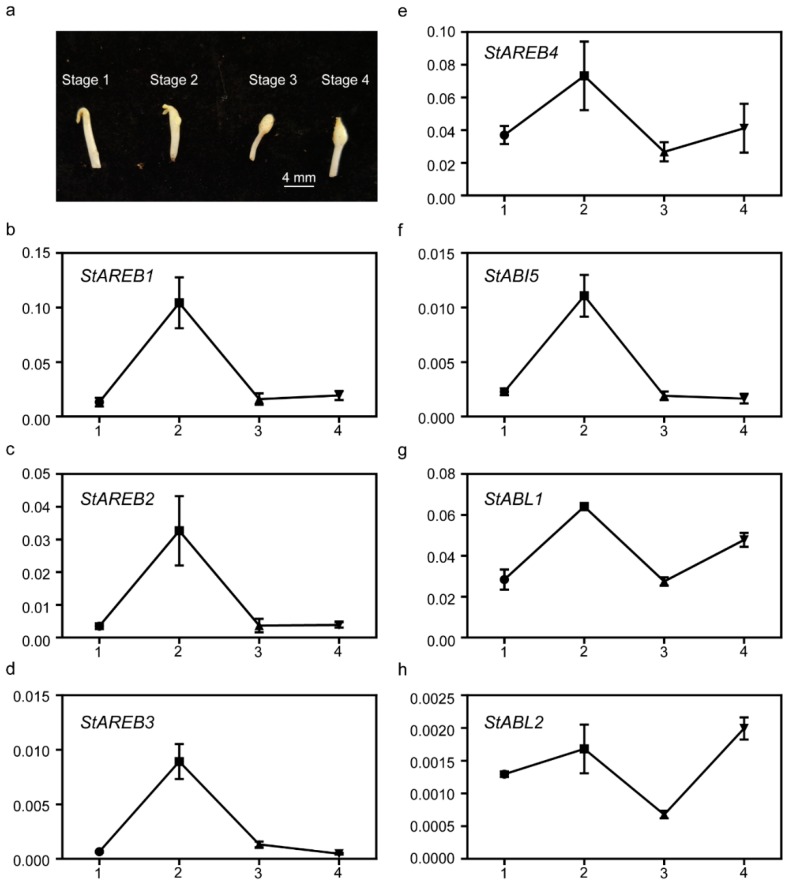
Expression profiles of potato *AREB/ABF/ABI5* members in distinct stages of stolon swelling. (**a**) Respective image showing distinct stages in the swelling process, ranging from no swelling (Stage 1) to prominent subapical swelling (Stage 4). (**b**–**h**) Graphs show the expression patterns of the potato *AREB/ABF/ABI5* members in the four different stages. The Y-axis is the relative expression levels after normalization with *ef1α*. The numbers 1, 2, 3, and 4 indicate the four stages shown in (**a**). Error bars represent the standard deviation (SD) of three biological replicates.

ABA is regarded as a promoting hormone in tuberization. The expression levels of potato AREB/ABF/ABI5 members in four distinct stages of the stolon swelling process were quantified (Figure 7). It was noticed that except for StABL2, which accumulated most transcripts in Stage 4, the expression levels of the other six AREB/ABF/ABI5 members were highest in Stage 2 (Figure 7). Besides that, StAREB1, StAREB2, StAREB3, and StABI5 exhibited similar expression patterns during the swelling process, showing specific accumulation of transcripts in Stage 2 (Figure 7), indicating that they may play developmental roles in this stage.

## 3. Discussion

As a well-characterized plant stress hormone, biosynthesis, perception, signal transduction and action of ABA in stress response, growth and development have been extensively investigated in the model plant *Arabidopsis* [2,4,41]. The identification of crucial components in ABA perception and signaling allowed the identification of downstream targets, i.e., the transcription factors, acting specifically in numerous ABA-dependent processes. AREB/ABF/ABI5 members served as key transcriptional regulators of ABA-dependent gene expression. These members play pivotal roles in developmental processes and the adaptation of plants to unfavorable environmental conditions [12,18,42,43]. However, the specific roles of the potato *AREB/ABF/ABI5* members in plant growth and stress response remain to be elucidated.

The present study characterized the structure, phylogenetic relationship, subcellular localization, transactivation activity, DNA binding activity, and expression profiles of whole AREB/ABF/ABI5 subfamily members in potato.

There are seven *AREB/ABF/ABI5* members in the potato genome, distributed in five chromosomes. Partial redundancy is often observed among members of a gene family, indicating that specific roles can be selected for duplicated genes while a shared set of functions is preserved [44]. Partially different gene structures, amino acid sequences, and induced expression patterns could also be noticed among the potato *AREB/ABF/ABI5* members, revealing that these genes might possess extensive functional redundancy, as well as independent functions.

In addition to highly conserved bZIP regions, four regions containing potential phosphorylation sites are also found to be conserved. These corresponding phosphorylation sites in *Arabidopsis* AREB1 have been demonstrated to be crucial for the regulation of activation [34]. StABF1, which was found to be identical to StAREB2 (Figure S3), has also been reported to be phosphorylated in response to ABA [33]. It would be of interest to explore the phosphorylation regulatory mechanism of potato AREB/ABF/ABI5 members in future work.

The *AREB/ABF/ABI5* members have been reported to be responsive to ABA and various environmental cues, although their induction patterns are varying [12,16,45]. In this study, seven potato *AREB/ABF/ABI5* members responded to ABA, drought, and high salinity in partially different patterns. Among them, *StAREB1*, *StAREB2*, and *StAREB4* are sharply upregulated by ABA, dehydration, and high salinity treatments (Figure 6), which coincided with a previous report [33]. AREB/ABFs have been reported to be widely used as marker genes for ABA signaling [46,47,48]. Considering these results, these three genes could also serve as ABA-response marker genes in potato. Recently, it was reported that *Arabidopsis* ABFs themselves are involved in mediating the rapid induction of their expression in response to exogenous ABA treatment, thus providing another layer of ABA regulation towards ABF proteins in addition to the well-characterized ABA-induced phosphorylation by SnRK2 protein kinases [49]. *Cis*-acting elements analysis of potato *AREB/ABF* genes found that *StAREB1*, *StAREB2/StABF1*, *StAREB3*, and *StAREB4* possessed ABRE in their promoter regions (Figure S4), implying that the rapid induction of their expression on exogenous ABA treatment might also be mediated by themselves.

There are various feasible ways of regulating AREB/ABF/ABI5 activities and, thus, controlling the expression of their target genes. As mentioned above, transcriptional regulation and phosphorylation may be two key factors regulating individual AREB/ABF/ABI5 member function. It was reported that *Arabidopsis* AREB/ABFs can form hetero- or homodimers to function in the nucleus [42], therefore, dimerization might be another way to regulate the activity of potato AREB/ABF/ABI5 proteins. Potato AREB/ABF/ABI5 members exhibited different transactivation activities in yeast cells (Figure 4), thus, several different combinations of AREB/ABF/ABI5 member dimers could be generated. Then a differential transcriptional activity could be provided to conduct a subtle regulation of their target genes.

The potato AREB/ABF/ABI5 subfamily members are nearly identical in their basic regions (Figure 2), thus, they appear to share conserved DNA binding activity. The in vitro binding assay confirmed that all of these proteins have an affinity to ABRE (Figure 5), which is highly conserved among ABA/stress-inducible promoters and involved in ABA inducibility in vivo [43]. This is well in line with previous studies that *Arabidopsis* ABF1 and ABF3 can bind ABRE in vitro [16]. A similar result was also reported, i.e., StABF1(StAREB2) had an affinity to ABRE [33].

In *Arabidopsis*, it was reported that three ABA- and water stress-responsive members of the AREB/ABF subfamily, AREB1/ABF2, AREB2/ABF4, and ABF3 are master transcription factors that cooperatively regulate ABRE-dependent ABA signaling involved in drought stress tolerance and require ABA for full activation [42]. In this study, it was found that *StAREB1*, *StAREB2*, and *StAREB4*, which belonged to the same phylogenetic clade of *Arabidopsis AREB1/ABF2*, *AREB2/ABF4*, *ABF3*, and *ABF1* (Figure 1), were dramatically induced by ABA, water deficit, and high salinity. Furthermore, these three potato AREB/ABF transcription factors behave similarly in terms of gene structure, nuclear localization, transactivation activity in yeast, and ABRE binding activity in vitro. Considering these data, it was reasonable to speculate that *StAREB1*, *StAREB2*, and *StAREB4* might function redundantly in regulating ABRE-dependent ABA signaling involved in osmotic stress. Nevertheless, the expression of *StAREB3*, another member of this clade (Figure 1), was at low levels compared with *StAREB1*, *StAREB2*, and *StAREB7* after treatment. Intriguingly, *Arabidopsis ABF1* has proved to be a functional homolog of *AREB1/ABF2*, *AREB2/ABF4*, and *ABF3* in ABA-dependent gene expression, despite lower expression levels of *ABF1* than those of the three *AREB/ABF*s [12]. Moreover, StAREB3 also exhibited similarity to StAREB1, StAREB2, and StAREB7 in nuclear localization, transactivation activity in yeast, and ABRE binding activity in vitro. Taken together with phylogenetic relationships among the four potato AREB/ABFs, it was rational to hypothesize that *StAREB3* might be a functional homolog of *StAREB1*, *StAREB2*, and *StAREB4*.

ABA has been demonstrated to promote tuberization [50]. In the present study, it was found that most *AREB/ABF/ABI5* genes were highly expressed in Stage 2 of the stolon swelling process (Figure 7), implying that they may play specific roles in this stage. Heterologous expression of the *Arabidopsis ABF4* gene in potato has been demonstrated to enhance tuberization through ABA-GA crosstalk regulation [51]. Therefore, it will be of interest to investigate the specific function of potato endogenous *AREB/ABF/ABI5* genes in tuberization.

## 4. Materials and Methods

### 4.1. Database Search and Sequence Retrieval

The potato AREB/ABF/ABI5 subfamily members were anchored by using the amino acid sequences of the known nine Arabidopsis AREB/ABF/ABI5 members [42] as a query to search against the Potato Genome Sequencing Consortium (PGSC, http://solanaceae.plantbiology.msu.edu/blast.shtml) database using the BLASTp programme. An E-value of 1e-20 was applied to reduce false positives. The potato AREB/ABF/ABI5 proteins were further confirmed by the Pfam database (http://pfam.xfam.org/).

### 4.2. Plant Materials and Treatments

For abiotic stress treatments, potato (*Solanum tuberosum* cv. E-potato 3) was grown in vitro at 22 °C day/18 °C night with a 16-h photoperiod for 2 weeks. The stress treatments were conducted as follows: ABA—in vitro plants were treated with 5 μM ABA in MS medium; drought stress—in vitro whole plants were pulled out from MS and placed in a well-controlled growth chamber; high salinity stress—in vitro plants were treated with 125 mM NaCl in MS medium. Then, roots and shoots were harvested together for immediate RNA extraction after 0, 6, and 24 h. For tuberization related analysis, E109, which shows a strict dependence on short days for tuber formation, was planted into plastic pots with a diameter of 10 cm (1 plant/pot) in a growth room with an 8 h light/16 h dark (LD) photoperiod and temperature 20 ± 2 °C. Tuberizing stolon tips that represented the four distinct stages of the stolon swelling process were taken 4 weeks after planting as previously described [52].

### 4.3. RNA Extraction and QUANTITATIVE Real-Time PCR

Total RNAs were extracted from frozen tissues using PLANTpure Universal RNA Kit (Aidlab, Beijing, China), and a post-treatment with DNase Digestion Kit (Aidlab, Beijing, China) was used to remove genomic DNA contaminants. Subsequently, the first-strand cDNA was synthesized using HiScript First Strand cDNA Synthesis Kit (Vazyme, Nanjing, China). Quantitative real-time PCR (qRT-PCR) was carried out with SYBR^®^ Green Real-Time PCR Master Mix (Vazyme) in a QuantStudio 6 Flex Real-Time PCR system (Thermo Fisher Scientific, Waltham, MA, USA ) following the fast cycling protocol. The potato *ef1α* (GenBank accession: AB061263) was selected as an internal reference gene for normalization. The specific primers pairs for each potato AREB/ABF/ABI5 members were designed using NCBI Primer-BLAST (https://www.ncbi.nlm.nih.gov/tools/primer-blast/). All primers are listed in Table S1.

### 4.4. Subcellular Localization Analysis

The full-length coding sequences (CDS) of potato *AREB/ABF/ABI5* genes with termination codon were amplified using potato cDNA as a template (refer to Table S1 for the primers used) and cloned into plasmid pH7LIC-N-eGFP with Exnase II (Vazyme, Nanjing, China) to produce the GFP-StAREB/ABF/ABI5s vectors for generating the GFP fused target protein in living cells. Subcellular localization of the GFP-StAREB/ABF/ABI5s was investigated at 60 h after infiltration by *Agrobacterium tumefaciens* GV3101 harboring the plasmid DNA with laser confocal fluorescence microscopy (Leica TCS-SPE, Wetzlar, Germany) in *Nicotiana benthamiana* epidermal cells.

### 4.5. Transactivation Activity Assays in Yeast

The CDS of *AREB/ABF/ABI5* genes were generated by amplification and cloned into pGBKT7 (Clontech, Palo Alto, CA, USA) to generate BD plasmid and primers, as shown in Supplemental Table S1. The plasmids were then transformed into AH109 yeast cells by the lithium acetate-mediated method. The transformed yeast strain was selected on SD/-Trp medium at 28 °C for 2 days. Positive transformants were then spotted onto fresh solid SD agar lacking Trp/His/Ade (SD/-Trp/-His/-Ade) to evaluate transactivation activity. The empty vector pGBKT7 was used as a negative control.

### 4.6. Protein Expression and Purification

The full-length *AREB/ABF/ABI5* genes or the truncated StAREB1-4 and StABI5 were amplified with specific primers listed in Table S1, and cloned in-frame in pET42b with SpeI and XhoI sites using Exnase II (Vazyme, Nanjing, China). All proteins were expressed in *Rosetta E. coli* (Novagen, Madison, WI, USA) by inducing with 0.2 mM isopropyl-β-d-thiogalactoside (IPTG) at 16 °C for 12–14 h. GST-tagged proteins were purified on Glutathione-Sepharose 4B (GS4B) resin (GE Healthcare, Pittsburgh, PA, USA) according to the manufacturer’s instructions.

### 4.7. Electrophoretic Mobility Shift Assays (EMSA)

Purified GST-tagged proteins were incubated with an FAM-labeled probe (ABRE) at 37 °C for 15 min to examine the binary complex. For the competition assay, competitors (unlabeled ABRE or mutant ABRE) were added in a 50- or 100-fold molar excess prior to the addition of the probe. The resulting reaction mixtures were subjected to electrophoresis of 1% agarose gel in 1× Tris/borate/EDTA buffer and were imaged with an Amersham Imager 600 (GE Healthcare, Pittsburgh, PA, USA).

## 5. Conclusions

In summary, a comprehensive analysis of the seven *AREB/ABF/ABI5* members in the potato genome was conducted in the current research. These genes shared high protein sequence similarity, especially in the bZIP region, indicating that they might possess overlapping roles in regulating gene expression. The nuclear location of seven AREB/ABF members was confirmed by the transient expression assay, suggesting that they might act as transcription factors to function in the nucleus. Transcriptional activation and in vitro DNA-binding assays demonstrated that some AREB/ABF/ABI5 shared both transcriptional activities and conserved DNA-binding activities, revealing that they may act as functional transcription factors in regulating the expression of ABA-response genes in potato plant. The transcription of *StAREB1*, *StAREB2*, and *StAREB4* can be strongly induced by ABA and osmotic stresses, which indicated that these three genes may have prominent functions in the ABA signaling pathway. Additionally, most *AREB/ABF/ABI5* genes were highly expressed in the stolon swelling process, implying that they may participate in the regulation of tuberization. All of these data will provide a solid foundation for the functional characterization of *AREB/ABF/ABI5* genes in potato, and further investigations on these genes are ongoing to uncover their precise biological functions.

## Figures and Tables

**Table 1:** General information for potato *AREB/ABF/ABI5* genes.

Gene	Gene ID	CDS	AA	MW	PI	Predicted Subcellular Localization
*StAREB1*	PGSC0003DMG400025889	1245	414	45.0	9.71	nucl: 14
*StAREB2*	PGSC0003DMG400008011	1362	453	48.8	9.50	nucl: 14
*StAREB3*	PGSC0003DMG400006211	1245	414	45.3	9.24	nucl: 14
*StAREB4*	PGSC0003DMG400022931	1101	366	40.1	8.95	nucl: 14
*StABI5*	PGSC0003DMG400002660	1287	428	46.4	8.92	nucl: 13
*StABL1*	PGSC0003DMG400028121	1044	347	37.9	7.91	nucl: 11.5, cyto_nucl: 6.5, chlo: 1
*StABL2*	PGSC0003DMG400007208	975	324	36.2	6.27	nucl: 13

Gene ID, PGSC IDs from Potato Genome Sequence Consortium database; CDS, length of coding sequence; AA, number of amino acids; PI, theoretical isoelectric point; MW, molecular weight, KDa; The subcellular location of potato AREB/ABF/ABI5 proteins was predicted using WoLFPSORT (http://www.genscript.com/psort/wolf_psort.html). Nucl, nucleus; Cyto, cytosol; Chlo, chloroplast. Testk used for kNN is 14.

## Supplementary Materials

Supplementary materials can be found at http://www.mdpi.com/1422-0067/20/2/311/s1. Figure S1: Schematic representation of the full length and truncation of AREB proteins used for protein expression, Figure S2: SDS-PAGE of recombinant StAREB1t, StAREB2t, StAREB3t, StAREB4t, StABI5t, StABL1, and StABL2, Figure S3: The protein sequences alignment between StAREB2 and StABF1, Figure S4: List of *cis* elements in *AREB* promoters of *Solanum tuberosum*, Table S1: Primers used in this research.

## Abbreviations

ABA	Abscisic acid
ABRE	ABA-responsive element
bZIP	Basic domain/leucine zipper
EMSA	Electrophoretic mobility shift assays
