# Supplementary material for: Genome-Wide Identification and Characterization of the AREB/ABF/ABI5 Subfamily Members from Solanum tuberosum
Source: Int J Mol Sci. 2019 Jan 14;20(2):311. doi: 10.3390/ijms20020311 (PMC6358972; doi:10.3390/ijms20020311)
Supplement: Supplementary file 1 [file ijms-20-00311-s001.pdf]

Genome-Wide Identification and Characterization of the AREB/ABF/ABI5 Subfamily Members from *Solanum tuberosum*

Supplementary Materials

Table 1. Primers used in this research.

Primer	Sequence (5′-3′)	Related Experiment
qRTStAREB1F	CCCCAAGCAAACAACAGTGG	q-RT-PCR
qRTStAREB1R	CATAACTCCGCCTTGAGCCA	q-RT-PCR
qRTStAREB2F	GGCAGTCATCTATCTATTCG	q-RT-PCR
qRTStAREB2R	CAACTCATCCATGTTCATTG	q-RT-PCR
qRTStABI5F	GAGGCAGTGGATTCCCACAA	q-RT-PCR
qRTStABI5R	CACTACTTGCCCGTAGCCAA	q-RT-PCR
qRTStAREB3F	TGGTGTTGGAGAGGGGAGAA	q-RT-PCR
qRTStAREB3R	ACTTCATCCACAGTCCGCTG	q-RT-PCR
qRTStABL1F	GGAACGGATTACGGGGTGTT	q-RT-PCR
qRTStABL1R	TCCTGCCACACCTGATCAAC	q-RT-PCR
qRTStABL2F	GGGGAAATGATTACGGGGCA	q-RT-PCR
qRTStABL2R	TCTTGCCACACCTCATCGAC	q-RT-PCR
qRTStAREB4F	CAAGCAACCCAAAACCCTGG	q-RT-PCR
qRTStAREB4R	TGCTGTGTTGAAGACGTGGT	q-RT-PCR
ef1α-RTF	ATTGGAAACGGATATGCTCCA	q-RT-PCR
ef1α-RTR	TCCTTACCTGAACGCCTGTCA	q-RT-PCR
GFPStAREB1F	ATTACGCCGAGGTCATGGGATCTTACATGAATTTC	Subcellular localization
GFPStAREB1R	TAGGGAAGAGGCTACCAAGGTCCTGTCAGTG	Subcellular localization
GFPStAREB2F	ATTACGCCGAGGTCATGGGGAGTAATTATCATTT	Subcellular localization
GFPStAREB2R	TAGGGAAGAGGTTACCATGGACCAGTTTGTG	Subcellular localization
GFPStABI5F	ATTACGCCGAGGTCATGGGAGTACCAGAATCAGA	Subcellular localization
GFPStABI5R	TAGGGAAGAGGTCAAGGGCAACTAAAGCTCC	Subcellular localization
GFPStAREB3F	ATTACGCCGAGGTCATGGGATCTTACTTGAACTT	Subcellular localization
GFPStAREB3R	TAGGGAAGAGGCTACCAAGGTCCTGTCAGTG	Subcellular localization
GFPStABL1F	ATTACGCCGAGGTCATGGGATCTCAGGGTGGTGG	Subcellular localization
GFPStABL1R	TAGGGAAGAGGTTAGACGGGCGCGGAGCTTG	Subcellular localization
GFPStABL2F	ATTACGCCGAGGTCATGGTAATACAAGGGATGGG	Subcellular localization
GFPStABL2R	TAGGGAAGAGGTCAGATAGGGCCTGAACTTG	Subcellular localization
GFPStAREB4F	ATTACGCCGAGGTCATGGGATCTTACCTGAACTT	Subcellular localization
GFPStAREB4R	TAGGGAAGAGGCTACCAAGGTCCCGTCACTG	Subcellular localization
BKStAREB1F	GGCCATGGAGGCCGAATTCATGGGATCTTACATGAATTTC	Transactivation activity
BKStAREB1R	GCGGCCGCTGCAGGTCGACGCTACCAAGGTCCTGTCAGTG	Transactivation activity
BKStAREB2F	GGCCATGGAGGCCGAATTCATGGGGAGTAATTATCATTT	Transactivation activity
BKStAREB2R	GCGGCCGCTGCAGGTCGACGTTACCATGGACCAGTTTGTG	Transactivation activity
BKStABI5F	GGCCATGGAGGCCGAATTCATGGGAGTACCAGAATCAGA	Transactivation activity
BKStABI5R	GCGGCCGCTGCAGGTCGACGTCAAGGGCAACTAAAGCTCC	Transactivation activity
BKStAREB3F	GGCCATGGAGGCCGAATTCATGGGATCTTACTTGAACTT	Transactivation activity
BKStAREB3R	GCGGCCGCTGCAGGTCGACGCTACCAAGGTCCTGTCAGTG	Transactivation activity
BKStABL1F	GGCCATGGAGGCCGAATTCATGGGATCTCAGGGTGGTGG	Transactivation activity

BKStABL1R	GCGGCCGCTGCAGGTCGACGTTAGACGGGCGCGGAGCTTG	Transactivation activity
BKStABL2F	GGCCATGGAGGCCGAATTCATGGTAATACAAGGGATGGG	Transactivation activity
BKStABL2R	GCGGCCGCTGCAGGTCGACGTCAGATAGGGCCTGAACTTG	Transactivation activity
BKStAREB4F	GGCCATGGAGGCCGAATTCATGGGATCTTACCTGAACTT	Transactivation activity
BKStAREB4R	GCGGCCGCTGCAGGTCGACGCTACCAAGGTCCCGTCACTG	Transactivation activity
42bStAREB1F	AATCGGATGGTTCAACTAGTATGGGATCTTACATGAATTTC	Protein expression
42bStAREB1R	CGAGTGCGGCCGCAAGCTTCTACCAAGGTCCTGTCAGTG	Protein expression
42bStAREB2F	AATCGGATGGTTCAACTAGTATGGGGAGTAATTATCATTT	Protein expression
42bStAREB2R	CGAGTGCGGCCGCAAGCTTTTACCATGGACCAGTTTGTG	Protein expression
42bStABI5F	AATCGGATGGTTCAACTAGTATGGGAGTACCAGAATCAGA	Protein expression
42bStABI5R	CGAGTGCGGCCGCAAGCTTTCAAGGGCAACTAAAGCTCC	Protein expression
42bStAREB3F	AATCGGATGGTTCAACTAGTATGGGATCTTACTTGAACTT	Protein expression
42bStAREB3R	CGAGTGCGGCCGCAAGCTTCTACCAAGGTCCTGTCAGTG	Protein expression
42bStABL1F	AATCGGATGGTTCAACTAGTATGGGATCTCAGGGTGGTGG	Protein expression
42bStABL1R	CGAGTGCGGCCGCAAGCTTTTAGACGGGCGCGGAGCTTG	Protein expression
42bStABL2F	AATCGGATGGTTCAACTAGTATGGTAATACAAGGGATGGG	Protein expression
42bStABL2R	CGAGTGCGGCCGCAAGCTTTCAGATAGGGCCTGAACTTG	Protein expression
42bStAREB4F	AATCGGATGGTTCAACTAGTATGGGATCTTACCTGAACTT	Protein expression
42bStAREB4R	CGAGTGCGGCCGCAAGCTTCTACCAAGGTCCCGTCACTG	Protein expression
42bStAREB1pF	AATCGGATGGTTCAACTAGTGATACATCGTCTCTTTCACC	Protein expression
42bStAREB2pF	AATCGGATGGTTCAACTAGTGATACACCTTCTGTATCTCC	Protein expression
42bStABI5pF	AATCGGATGGTTCAACTAGTGATAATGTGGGCCAATACGG	Protein expression
42bStAREB3pF	AATCGGATGGTTCAACTAGTAACAGGCCATCTCCTTCCCC	Protein expression
42bStAREB4pF	AATCGGATGGTTCAACTAGTGATACATCTTCTCTTTCACC	Protein expression

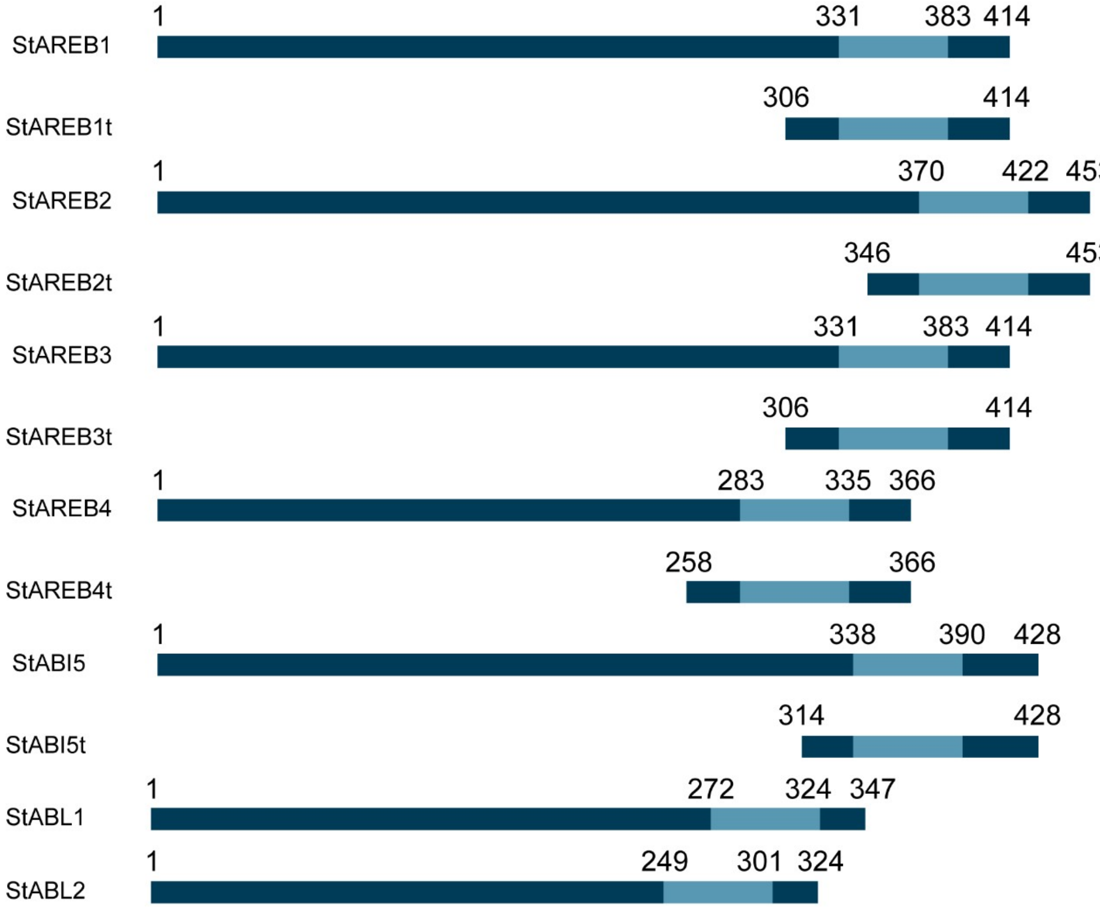

Figure 1. Schematic representation of the full length and truncation of AREB/ABF/ABI5 proteins used for protein expression. The conserved basic domain/leucine zipper (bZIP) domains are indicated by red color. The numbers above designate the amino acid positions of the regions.

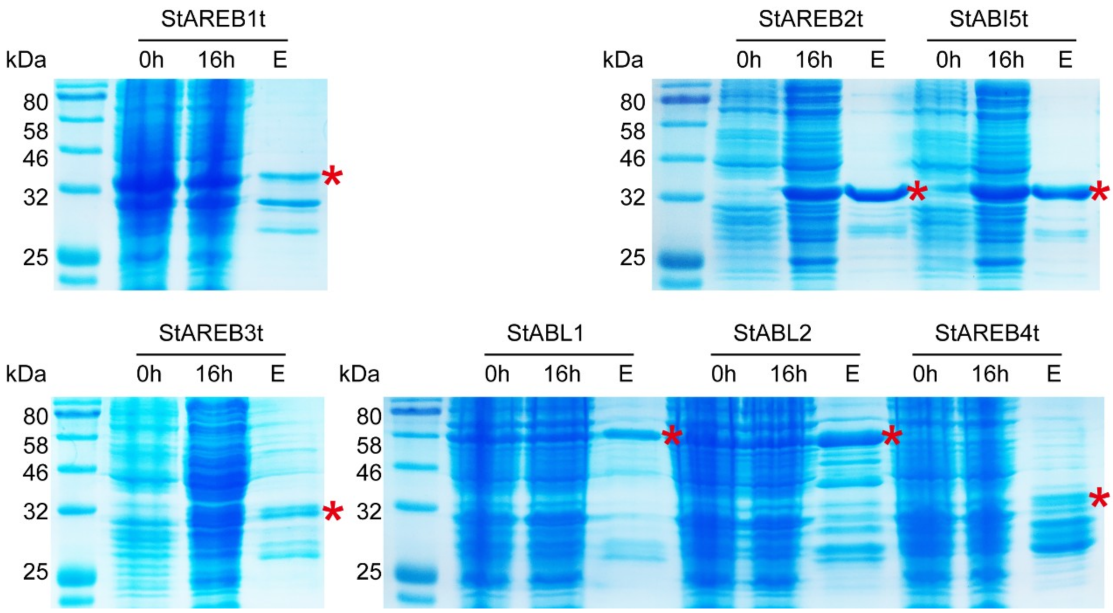

Figure 2. SDS-PAGE of recombinant StAREB1t, StAREB2t, StAREB3t, StAREB4t, StABI5, StABL1, and StABL2. The recombinant proteins containing N-terminal GST-tags were expressed in *Escherichia coli* after induction with 0.4 mM IPTG. Extracts of *E. coli* BL21 before (0 h) and after induction (16 °C for 16 h) and the proteins purified with GST affinity resin (E) are shown after SDS-PAGE.

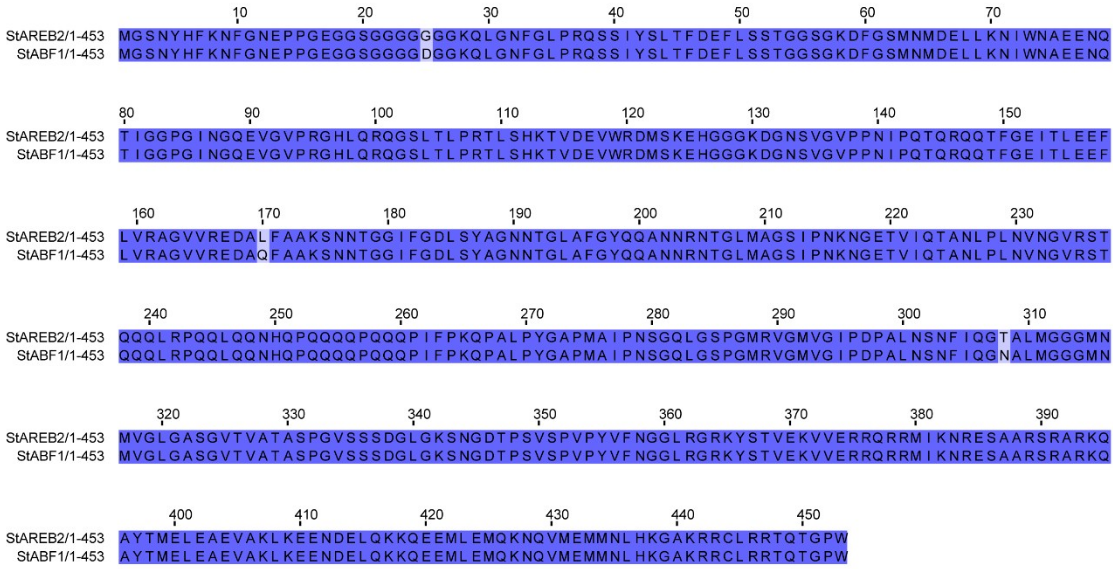

Figure 3. The protein sequences alignment between StAREB2 and StABF1.

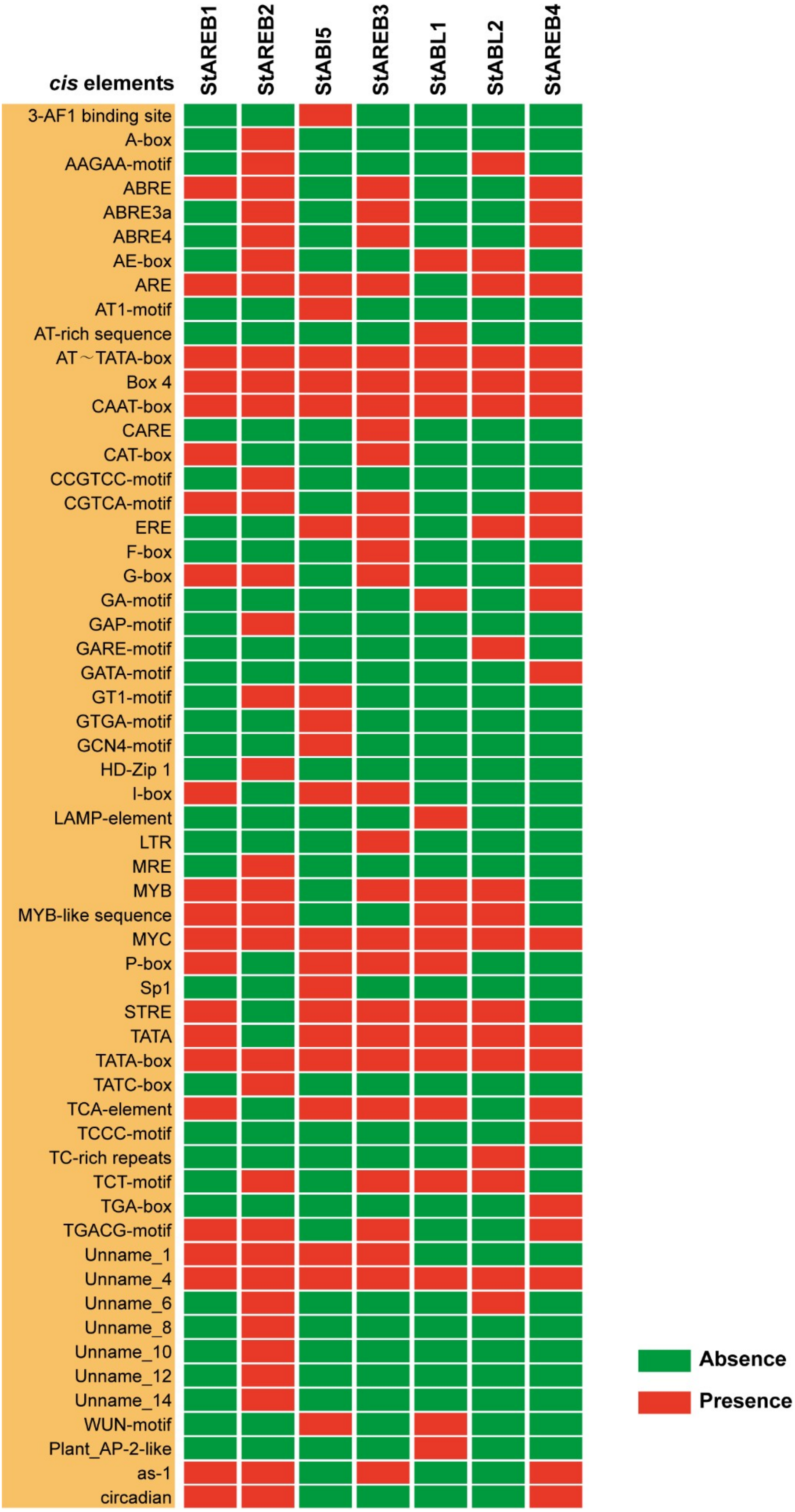

Figure 4. List of *cis* elements in *AREB*/*ABF*/*ABI5* promoters of *Solanum tuberosum*.
